# Bibliographical Notices

**Published:** 1849-01

**Authors:** 


### BIBLIOGRAPHICAL NOTICES.

*Essays on Infant Therapeutics: To which are added Observations on Ergot, and an account of the Origin of the use of Mercury in inflammatory complaints.* By JOHN B. BECK, M. D., Professor of Materia Medica and Medical Jurisprudence, in the College of Physicians and Surgeons of the University of the State of New York, &c., &c. 12mo. pp. 117. W. E. Dean, New York, 1849.

These Essays have appeared, at different times, in several of our Medical Journals; and from their favourable reception by the profession, the author has been induced to republish them in the present form. By doing so, he has conferred a substantial benefit on the profession, and promoted the cause of humanity. Many physicians are either not aware, or not sufficiently mindful of the great susceptibilities of the constitutions of very young subjects to the effects of what are called heroic remedies, and of course, the unprofessional who administer such without the physician's knowledge, often do incalculable mischief. To both of these classes of prescribers, the Infant Therapeutics of Dr. Beck will prove an instructive monitor.

The first Essay is *On the effects of Opium on the young subject.* "With regard to the effects of opium on young subjects," observes the author, "there are two facts which seem to be well established. The *first* is, that it acts with much *greater energy* on the infant than it does on the adult; the *second* is, that it is more *uncertain* in its action on the infant than the adult. It is in consequence of these peculiarities attending its operation on the infant, that even the smallest quantities have not unfrequently produced the most unexpected and even fatal results." Cases are cited from various authors in proof of these positions.

"The causes of this (difference between the infant and adult) would seem to be the following:

"In the *first place*, the great difference in the physical organization of the *infant* and the *adult.* In the young subject the brain and nervous system are much more impressible, and the slightest causes, as we know, will sometimes derange them. Besides, in the infant, the circulation is more rapid—there is a greater proportionate quantity of blood circulating in the brain, and hence a much greater tendency to cerebral determinations. From these peculiarities in the organization of the infant, it happens that convulsions are so much more common in the early periods of life. Thus, for example, the irritation of teething—of worms or crude matters in the intestines, is frequently followed by convulsions. Intermittent fever, which in the adult, commences with a chill, in the child is frequently ushered in by a convulsion. Scarlet fever, too, in the child, not unfrequently commences with a convulsion, while in the adult I have never witnessed such an occurrence. Now, with such peculiar predispositions characterizing the system in infancy, it may readily be conceived how it is that such an article as opium should act with more power at that period than in after life.

"In the *second place*, the difference in the *temperament* or *constitution* of infants. In the adult, we know as a matter of fact, that opium differs greatly in its effects in different constitutions. Thus, as a general rule, the sanguine temperament does not appear to bear the use of this drug as well as the melancholic or the nervous. In the former, it is much more apt to produce cerebral disturbance, and in large doses is more likely to prove injurious. Now, infants differ from one another, as much, if not more, than adults, in these peculiarities of constitution. and, as a matter of course, the difference in the effects of this article must be greater. Besides, as these peculiarities and differences can only be detected by actual experience, and as we cannot of necessity have the same benefit of experience in the case of infants, it is obvious that the difficulty of justly appreciating the action of this drug on the infant must be greatly increased. A greater or less degree of uncertainty, therefore, must necessarily from this cause attend its use in the early periods of life.

"In the *third place*, the actual state of the system as to disease. There is no circumstance which modifies the effect of opium in so great a degree as this. In the adult, we see this continually. In some conditions of the system, even small doses produce the most unpleasant effects, while in other conditions, immense quantities may be given with little or no effect; thus, for example, when severe pain or spasm is present, quantities of this article can be borne, which under other circumstances would prove exceedingly injurious."

From these peculiarities of the action of opium, the author very justly remarks "that its use should *be avoided as much as possible*

in the young subject,"—that is, that it should not "be used without a strong and manifest necessity," and of this necessity, a well instructed physician is of course the only proper judge.

"Great caution should be exercised in the *form* in which it is administered. No preparation should ever be used, which *is not of a known and determined strength;*" and "in very young subjects, we should never begin the use of this article, except in *very small doses.*"

The author prefers, as "the best preparations for children," *laudanum*, *paregoric*, and *Dover's powder*. The formulæ for these are in the Dispensatories, and when properly prepared, they are of uniform strength. He objects to the syrup of poppies, Dalby's carminative, Godfrey's cordial, &c., because of their *uncertain* strength. Even with regard to the preparations recommended, much caution is necessary from the very vague directions given by authoritative writers for their employment in the treatment of particular diseases.

Essay II. is *On Emetics.* Professor Beck maintains that, in infancy, from anatomical and physiological peculiarities, vomiting is more readily effected than in the adult, and that, therefore, the milder articles should be employed, and those of an active and energetic character, which produce much nausea, as the antimonials, avoided. As a general rule, this is undoubtedly correct. Every practitioner of experience must have witnessed cases where, from the frequent or long continued use of antimonials, either in emetic or nauseating doses, in hooping cough, scarlet fever, and measles, alarming sedation has been induced, from which no ordinary restoratives could snatch the sufferers.

Essay III. is *On the effects of Mercury on the young subject.* In this essay the same cautious spirit is manifested as in the preceding. The latitude within which our author would allow of the use of mercury in the young subject, is greatly restricted in comparison with the general custom—more restricted, perhaps, than is always necessary. He thinks that because in young subjects it is not so liable to salivate, the temptation to employ it is the greater and leads to bad consequences. Contrary to general opinion, salivation from the use of mercury does sometimes occur before the third year, and even in younger subjects, and then it is most disastrous. "*Although mercury so seldom salivates infants,*

*yet, notwithstanding this, it cannot be doubted that it affects the system profoundly, and even more so proportionally than it does the adult.*" "A single large dose of calomel," he remarks, "will cause nausea and relaxation, and sometimes unpleasant prostration, while if it be given in smaller doses and repeated frequently, it will occasion irritation of the intestines, and general disturbance of the vascular and nervous system. In the former case, acting as a profound sedative, and in the latter as a stimulant, or rather irritant."

Although it be true that large doses of calomel are capable of producing profound sedation, both in the young and the old, and therefore should not be needlessly had recourse to, it is important to avoid the other extreme, of withholding the remedy in many cases in which there is no effectual substitute for it. The instances in which it occasions salivation in those under *two* years old are so rare, and the cases in which it may be employed advantageously, both as a cathartic and alterative, so numerous, that we should not hesitate to prescribe it when called for with as little dread as any other medicine. Simply as a cathartic, without any other object than merely to evacuate the bowels, it never should be administered, *because it always does exert a wider influence;* but it is in consequence of this extensive action that it is frequently of such signal benefit.

Essay IV. Treats *of the effects of blisters on the young subject.* The peculiarities attending the operation of blisters on the young subject are the following:

1st. They produce their effects in a shorter time than they do in the adult.

2d. The local inflammation produced by a blister is greater in the young subject than the adult.

3d. Blisters are more apt to be followed by the injurious consequences of inflammation, such as ulceration, gangrene, and even death.

4th. The constitutional excitement produced by blisters is generally greater in young subjects than in the adult.

Numerous cases are cited by the author, illustrative of these points.

The author, from the effects of blisters, thinks it follows that they may be rendered more efficient as a means of cure in their diseases than in adults, under similar circumstances.

The essay contains many sound observations of great practical importance on the use of these powerful agents—with a greater leaning to their employment, however, it seems to us, than accords with our own experience of their expediency.

The 5th Essay is *on "the effects of blood-letting on the young subject."*

The author asserts that the young subject does not bear the loss of blood in considerable quantities, nor the repetition of the operation, so well as the adult, and that the nervous system in them is more powerfully affected by it.

We regard this essay as one of the most important in the volume, and eminently practical in its scope, and sound in doctrine. Indeed, all are of that character; nor do we know of any publication, of recent date, in which so much information and counsel of great value to the young practitioner, on the subjects of which it treats, is to be found as in this small volume of Professor Beck.

---

*An Introductory Lecture, delivered to the Class of Institutes of Medicine in Jefferson Medical College, October* 19, 1848. By Robley Dunglison, M. D. Published by the Class.

Professor Dunglison is one of our most attractive medical lecturers, and the lecture before us fully sustains that reputation. After introducing himself to his class in his happiest style, we are pleased to find him choosing, as the subject of his discourse, some point connected with the study of Physiology. This we conceive to be the true object of an introductory lecture, though it has been, and still is, too common for Professors, on such occasions, not only to say little or nothing about the subject they propose teaching, but to wander from the domain of medicine in search of material from some other department of science. This plan may be pleasant enough to the writer, but cannot prove instructive to the medical student; and we fear that many lectures are written rather to catch the public applause than to convey useful general instruction in the various branches of medical science.

Dr. Dunglison, in his lecture, commences with a general survey of the rise and progress of Histology, or the minute anatomy of the tissues of the body. One of the earliest objects of the Histologists was, to ascertain "whether all organized bodies, resembling each other as they do, so highly in their mode of development, might not by ultimate analysis be found to originate in the same manner." To the early researches of the Edwardses, of Raspail, and more recently of Schwann and Schleiden, we are indebted for the discovery of the beautiful theory of cell development, by which it has been proved that "the elementary parts of all tissues are formed of cells in an analogous though very diversified manner, so that it may be asserted, *that there is one universal principle of development for the elementary parts of organisms, however different, and that this principle is the formation of cells.* A structureless substance—as the liquid germ of the vegetable, and the liquor sanguinis of the animal—is present in the first instance, which lies either around or in the interior of cells already existing, and cells are formed in it, in accordance with certain laws; which cells become developed in various ways into the elementary parts of organisms." But these cells, whether of animal or vegetable origin, besides resembling each other in form, &c., are found to be identical in chemical composition—a fact of the highest importance in the dietetic management of diseases.

The lecturer next proceeds to speak of the "impulse seated in the living ovum and seed, which, under favourable circumstances, leads to the development of an organised body, according to a definite plan and arrangement." Of the nature of this impulse we are entirely ignorant, but enough is known of the mode in which, under its influence, every organised tissue is developed, to prove that its existence is anterior to the formation of bloodvessels and of nerve matter, and consequently these cannot be regarded as identical with that vital principle with which the cells seem to be endowed. It appears, moreover, that these cells are possessed of an instinctive force, capable of repairing, to a certain extent, injuries inflicted upon the animal or vegetable structure. All these points are appropriately illustrated by the lecturer; but for these, and some interesting remarks upon the immense distances to which animalcules and cryptogamic sporules may be transported, thus probably becoming a prolific source of disease, we would refer the reader to the lecture itself, which he will find well written and highly instructive.

---

The above is the only Introductory that was received in time for notice in this number. Several, that have come to hand since, will be noticed in the February number.

---

*Medical Lexicon of Modern Terminology; being a complete Vocabulary of Definitions, including all the technical terms employed by Writers and Teachers of Medical Science at the present day, and comprising several hundreds of words not found in any other Dictionary. Designed for the use of Students and Practitioners. Second edition, greatly enlarged.* By D. MEREDITH REESE, M. D., LL. D., Resident Physician of Bellevue Hospital; Editor of Cooper's Surgical Dictionary, &c. 24mo. pp. 230. New York: Samuel S. & William Wood. 1848.

The ready sale of the first edition of this little work is sufficient evidence of its acceptableness to those for whom it was especially intended. The demand for a second edition rendered it "imperative upon the author to correct, improve and enlarge the work, before again committing it to the press." It is with regret we notice that this commendable intention has not been carried out, and that many, if not all the glaring defects of the previous edition, as pointed out in a notice published in this Journal, in February, 1845, are still allowed to stand unchanged.

In addition to those then mentioned, many others might be cited which meet the eye on almost every page, which, if not positive errors, are, at least, inaccuracies. Such, for instance, as ACCELERATOR URINÆ: "*a muscle of the bladder.*" ABDOMINAL CAVITY: "*the sac of the peritoneum is strictly so called, excluding the kidneys and pelvic viscera.*" According to this definition, then, the peritoneal sac and the abdominal cavity are the same, and the kidneys are not in the cavity of the abdomen! Opening again at random, we find PES ANSERINUS: "*a goose's foot, distribution of a plexus of nerves on the side of the face; seat of the tic*

*dolouroux.*" The student is allowed to choose between *the portio dura of the seventh*, and *the branches of the fifth*, as to which is the *goose's foot.* If he be aware which forms the *pes anserinus*, he is misinformed as to which is the seat of neuralgia. Again; SEMIMEMBRANOSUS: "*muscle of the thigh.*" SEMITENDENOSUS: "*muscle of the leg*"*!* Numerous examples of a similar description might be mentioned, but these are perhaps sufficient to indicate the character of the work in regard to accuracy of definition.

The author further states, in the preface to the second edition, that the changes and improvements which are perennial in every branch of the healing art, are of necessity suggestive of new technicalities, "hence the office of the humble lexicographer imposes upon him the duty of collecting and defining these in every reprint of his book; a task which," it is complacently said, "has been performed in the present instance, as it is hoped, to the full extent desirable in such a dictionary." Under this assurance, we turned confidently to look for the terminology of the world-wide cell-doctrine, but found nothing to assist the student in the comprehension of this abstruse subject.

It is with pain that we are obliged to subscribe to the opinion previously expressed in regard to the first edition, and to regret that a work whose character and object are so manifestly useful to the student, should be so marred by the glaring inaccuracies and defects designated in this and the previous notice.

---

*A Text-Book of Practical Anatomy.* By ROBERT HARRISON, M. D. M. R. I. A., Fellow of the Royal Colleges of Surgeons of Ireland and England; Professor of Anatomy and Surgery of the University of Dublin, etc. With additions by an American Physician. With numerous illustrations. 8vo. pp. 720. Samuel S. & William Wood. New York, 1848.

This, we perceive, is our old companion, "*The Dublin Dissector,*" under a new name; and with additions, too, by an American Physician. Who the "American Physician" is, what are his qualifications, and what are the additions which he has made, we are not informed, any more than of the reason why his name is not on the title page as the lawful claimant of his share of the publication. To these omissions we have decided objections. They leave the reader in doubt as to what belongs to the author, and, so far, are unjust to him as well as to the Annotator, who, if his additions are really valuable, is deprived of his due by the suppression of his name. Neither do we see the propriety of a change in the title of the book. The old name of "Dublin Dissector," by which every body knew it, is just as expressive as that which the "American publishers" have deemed "the more appropriate title," beside the discourtesy of christening an author's work by a title which he has not seen fit to bestow upon it. It is a liberty, we think, which is not warrantable under any circumstance.

The Dublin Dissector has long held, and deservedly, a high rank among the hand-books for the guidance of the student of anatomy in the dissecting room, and the illustrations contained in the latter editions much enhance its value; but for the general study of the science, it is defective from the total omission of histology.

The typographical execution of the volume before us is very creditable to the publishers, but the illustrations are shockingly bad. To those who are accustomed to look upon specimens of the kind executed in London or Philadelphia, and sometimes in New York publications, the contrast is anything but agreeable.

